# Understanding organ donation refusal in the next of kin: a fifteen-year retrospective study in ten thousand potential donors

**DOI:** 10.1186/s44158-025-00282-7

**Published:** 2025-10-09

**Authors:** Stefano Marelli, Lorenzo Querci, Federico Pozzi, Cristiana Cipolla, Giuseppe Piccolo, Marco Sacchi, Tullia De Feo, Massimo Cardillo, Arturo Chieregato

**Affiliations:** 1 School of Medicine and Surgery, University of Milano-Bicocca, Milan, Italy; 2 Ospedale Niguarda Ca’ Granda, Neurocritical Care Unit, Milan, Italy; 3 Coordinamento Regionale del Procurement Di Organi e Tessuti - AREU Lombardia, Milan, Italy; 4 Coordinamento Regionale Trapianti, Direzione Generale Welfare, Milan, Regione Lombardia Italy; 5 S.C. Trapianti-Lombardia, North Italian Transplant Program (NITp), Fond. IRCCS Ca’ Granda Ospedale Maggiore Policlinico Di Milano, Milan, Italy

**Keywords:** Organ donation, Geographic origin, Intensive Care Units variability, Refusal, ICU-specific factors, Donor-specific factors

## Abstract

**Introduction:**

Refusal of organ donation is influenced by a range of interconnected factors spanning donor characteristics, family dynamics, and intensive care unit (ICU) practices. This study explores the impact of donor or centre-related variables on organ donation refusal rates in Italian ICUs, among potential donors without expressed will.

**Methods:**

We conducted a retrospective analysis of 12,930 potential donors registered in the North Italian Transplant Program registry from 01/01/2010 to 31/03/2025. A linear mixed-effects model was applied to account for donor characteristics (age, timing and cause of death, geographic origin) and ICU-level variability with refusal of organ donation.

**Results:**

In multivariate analysis geographic origin was an independent predictor of refusal – particularly for donors from North Africa and Middle East (OR 9.59, IQR 6.25 – 14.72; *p*-value < 0.001), Asia (OR 7.76, IQR 5.69—10.57; *p*-value < 0.001), Africa (OR 6.81, IQR 4.44 – 10.45; *p*-value < 0.001) and eastern European (OR 2.65, IQR 2.17 – 3.23; *p*-value < 0.001). Also, time from event to death over 48 h was associated with higher refusal rate (OR 3.37, IQR 2.52 – 4.50, *p*-value < 0.001). In contrast, traumatic brain injury (OR 0.85, IQR 0.74 – 0.98; *p*-value 0.023) was protective. Finally, inter-ICU variability had a significant impact on refusal rates, as indicated by a Median Odds Ratio of 1.38. However, the multivariate model demonstrated weak predictive ability for organ donation refusal (AUC = 0.66).

**Conclusions:**

This study identifies several factors independently associated with organ donation refusal. However, the overall predictive ability based on available variables remains limited. To enable individualized interventions and effectively reduce refusal rates, more comprehensive and prospective data collection is warranted.

**Supplementary Information:**

The online version contains supplementary material available at 10.1186/s44158-025-00282-7.

## Introduction

Generally, when individuals have not explicitly expressed their consent to organ donation during their lifetime, the responsibility to authorize or refuse donation falls to their next of kin. Given that in many countries a large proportion of the population has not registered a decision, the role of families becomes pivotal in the donation process. In Italy, for instance, over 70% of individuals are estimated to remain unregistered in the national donor registry [1]. Family authorization for organ donation in the absence of explicit consent by deceased, remains a pivotal step in the donation process. Literature on attitudes toward organ donation highlights diverse global perspectives influenced by individuals and contextual factors. Potential donor’s geographic origin it’s known to be influential [2–10], however it does not act in isolation; in fact, a growing body of research suggests that both cognitive and non-cognitive determinants – such as knowledge, cultural and religious belief, language barriers, trust in the healthcare system – contribute to shaping willingness to donate [11–13]. At the same time, extrinsic factors – including ICU protocols, staff training in cultural competence, institutional resources, and the quality of interactions between healthcare professionals and families – play a decisive role [14–17]. Importantly, decision to authorize organ donation in critical care depend on both staff actions and family decisions; they are inextricably intertwined, and consent often emerges from a relational process marked by emotional, ethical and communicative complexity [10, 14, 17].

This study analyses the most influential factors in shaping family decisions in a prospective database collecting data from Italian ICUs. The primary aim is to provide a deeper understanding of what facilitates or hinders the expression of a positive donation will in the absence of prior consent.

## Materials and methods

### Clinical trial number

Not applicable.

### Consent to participate declaration

Informed consent was not applicable, as the study involved deceased individuals (potential organ donors). According to applicable regulations and ethical standards, including the Declaration of Helsinki and GCP 2025 guidelines, the study was conducted using anonymized data and was approved by the Internal Review Board, which waived the requirement for informed consent due to the retrospective nature and the involvement of deceased persons only.

### Ethics approval declaration

Human Ethics and Consent to Participate declarations: not applicable.

### Study design and population

This study was performed as a retrospective analysis and reported according to STROBE standards for observational studies. We consecutively enrolled all potential deceased donors entered from 01/01/2010 to 31/03/2025 in the North Italian Transplant Program (NITp) registry. Covariates available in the database are reported in online Supplementary Material.

North Italian Transplant Program is an interregional organization serving six regions of northern Italy (an area of approximately 20 million inhabitants, with 130 hospitals active in donor procurement, and 35 transplant centres) and is responsible for coordinating the donation process, transplant safety evaluation, organ allocation, and donor-recipient immunological matching.

Among the potential donor-related confounders we evaluated a) age, b) sex, c) the cause of death, d) the birth nation, e) amount of time between event and death, f) the ICU in whom the donor was admitted.

The total number of registered potential donors was 12,930. We excluded the following patients: 482 donors (3.7%) for whom donation wishes were not requested due to evident ineligibility for donation; 4 donors (< 0.01%) with missing data on donation wishes and place of birth; 648 donors (5.5%) who underwent donation via DCD (donation after circulatory death); and finally, 1,506 donors (12.8%) who had formally expressed their donation wishes during their lifetime, obtaining a final study population of 10,254. (Supplementary Material, Fig. 1S).

The methodology for grouping birth nations into macro groups to produce nine macro areas of donor geographic origin is described in Supplementary Material, Fig. 4S (Africa, Asia, Eastern Europe, Western Europe, North Africa and the Middle East, North America, Oceania, and South America).

Since the majority of the studied donor population was born in Italy, we also considered their geographical variability within the country, using the commonly accepted division into north (Val d’Aosta, Piemonte, Liguria, Lombardia, Emilia Romagna, Trentino Alto Adige, Veneto, Friuli Venezia Giulia), central (Toscana, Marche, Umbria, Lazio) and south Italy (Abruzzo, Molise, Campania, Puglia, Calabria, Sardegna, Sicilia), based on place of birth.

Among the potential centre-related confounders, we evaluated the number of potential donors recruited per ICU, while the ICU itself was considered as a potential proxy for level of expertise.

### Statistical analysis

We described continuous variables using the median and interquartile range (IQR), while we summarized categorical variables with frequencies and percentages. We performed univariate analyses using non-parametric tests: the Mann–Whitney U test to compare continuous variables between two independent groups, the Fisher's exact test to compare the frequencies of categorical variables, and ANOVA for multiple categorical outcomes. The ICU centre is a multiple variable, so we used a linear mixed-effects model to assess the effect on the model. This model allows for the handling of within-centre correlations and the inclusion of random effects. We selected independent variables included in the model based on clinical relevance and on the results of the univariate analysis. We quantified among-centre differences in refusal of organ donation using the median odds ratio (MOR) after adjusted generalized linear mixed modelling, with ICU centre, ICU volume, and year of donation as random effects. MOR referred to the odds of refusal of organ donation between two randomly selected centres for patients with the same covariates. A MOR higher than 1 denoted higher variability among centres. We used linear regression to assess the effect of centre volume on the refusal rate. We conducted all statistical analyses using R software (version 4.5) and used the ‘lme4’ package for the analysis with linear mixed-effects models. Finally, we evaluated the predictive performance of the final model using the area under the receiver operating characteristic curve (AUC), and model robustness was tested via internal validation with bootstrapping.

## Results

### Case material

After applying the exclusion criteria, the final study population consisted of 10,254 (127 ICUs) potential donors (Table 1). The majority were born in Italy (*n* = 9,038; 88%). Eastern Europe represented the next largest area (*n* = 454; 4.4%), followed by Asia (*n* = 213; 2.1%) and Western Europe (*n* = 182; 1.8%). Other geographic areas included South America (*n* = 132; 1.3%), North Africa and the Middle East (*n* = 116; 1.1%), Africa (*n* = 101; 1.0%), North America (*n* = 12; 0.1%), and Oceania (*n* = 6; < 0.1%).

**Table 1:** Donation attitudes over time and associated factors

			Univariate analysis				Multivariate analysis**			
			Total, *N* (%)	Permission	Refusal	*p*-value	OR	95% CI	*p*-value	Missing Data
Patient’s variables	Gender	Female	4560 (44%)	3245 (71%)	1315 (29%)	0.035	Ref			
		Male	5694 (56%)	4159 (73%)	1535 (27%)		0.87	0.79 – 0.95	0.002	0
	Age (years)	Median (IQR)	63 (49, 74)	64 (50, 74)	62 (47, 74)	0.004	1.01*	0.98 – 1.04	0.513	20
	Geographic origin	Italy	9038 (88%)	6792 (75%)	2246 (25%)		Ref			
		Western & Northern European	182 (1.8%)	135 (74%)	47 (26%)	< 0.001	1.13	0.81 – 1.59	0.473	0
		African	101 (1.0%)	33 (33%)	68 (67%)		6.81	4.44 – 10.45	< 0.001	
		Asian	213 (2.1%)	61 (29%)	152 (71%)		7.76	5.69 – 10.57	< 0.001	
		Eastern European	454 (4.4%)	249 (55%)	205 (45%)		2.65	2.17 – 3.23	< 0.001	
		North African & Middle Eastern	116 (1.1%)	30 (26%)	86 (74%)		9.59	6.25 – 14.72	< 0.001	
		North American	12 (0.1%)	10 (83%)	2 (17%)		0.72	0.16 – 3.34	0.677	
		Oceanian	6 (< 0.1%)	3 (50%)	3 (50%)		2.75	0.52 – 14.49	0.234	
		South American	138 (1.3%)	91 (66%)	47 (34%)		1.30	0.89 – 1.90	0.177	
	Italian Born	North Italian born	7046/9038 (78.0%)	5419 (77%)	1627 (23%)	0.004	Ref			
		Centre Italian born	951/9038 (10.5%)	683 (75%)	268 (25%)		1.34	1.08 – 1.65	0.008	
		South Italian born	1041/9038 (11.5%)	690 (66%)	351 (34%)		1.61	1.39 – 1.86	< 0.001	
	Cause of death	Cerebral haemorrhage	5660 (55%)	4090 (72%)	1570 (28%)		Ref			
		Other	358 (3.5%)	243 (68%)	115 (32%)	0.003	1.13	0.88 – 1.45	0.348	0
		Head trauma	1632 (16%)	1233 (76%)	399 (24%)		0.85	0.74 – 0.98	0.023	
		Post-anoxic encephalopathy	1628 (16%)	1151 (71%)	477 (29%)		1.00	0.87 – 1.15	0.964	
		Ischemic stroke	976 (9.5%)	687 (70%)	289 (30%)		1.07	0.92 – 1.25	0.381	
	Time from event to Death	≤ 48 h	10046 (98%)	7311 (73%)	2735 (27%)	< 0.001	Ref			0
		> 48 h	208 (2%)	93 (45%)	115 (55%)		3.37	2.52 – 4.50	< 0.001	
ICU centre volume (*n*)		Median (IQR)	257 (91, 390)	257 (91, 390)	257 (95, 388)	0.140	/			

Data are displayed as Number (%) or median (IQR). This table shows donation attitude towards donation trends through the studied years, demographics, clinical variables, expression of patient will and ICU performance in relation to approval of organ donation after death

It also displays *p*-values from the univariate analysis and OR (95% CI) with relative *p*-values from the multivariate analysis

^*^Age in multivariate analysis transformed into decades to improve model performance and interpretation but did not show a significant association with refusal

^**^Multivariate model: Dependent variable: refusal to donate an organ; Independent variables: geographic origin, age, gender, cause of death, time from event to death; random effect: ICU centre

The population was slightly male-predominant (56% male vs. 44% female). The median age was 63 years (IQR 49–74). Most individuals (98%) had a hospital stay of 48 h or less prior to brain death assessment.

Cerebral haemorrhage was the most common cause of death, accounting for 55% of cases (*n* = 5,660), followed by traumatic brain injury (TBI) (*n* = 1,632; 16%) and post-anoxic encephalopathy (*n* = 1,628; 16%). Ischemic stroke was identified in 9.5% of donors (*n* = 976). Less common causes, grouped under “other” (*n* = 358; 3.5%), included neoplasms (86/358, 24%), infections (165/358, 46%), gunshot wound (42/358, 12%), and intracranial hypertension due to causes other than those previously listed (65/358, 18%). Time from event to death was ≤ 48 h in 10,046 cases (98%) and > 48 h in 208 cases (2%). Donors were identified across 127 intensive care units, with a median centre volume of 257 cases (interquartile range [IQR] 91–390).

### Univariate analysis

Analysis showed significant variability among the median age values, with a statistically increased refusal rate in younger patients (62 years against 64 years; *p*-value 0.004). Although this result is statistically significant it does not appear to be relevant due to the small difference in clinical characteristics in the donation management process. Male gender appears to be associated with a significantly higher approval for organ donation (27% refusal in males vs 29% in females; *p*-value 0.035).

Cause of death was significantly associated with the organ donation consent rate (*p* = 0.003), with refusal rates ranging from 24% (*n* = 399/1632) among patients who died from head trauma to 30% (*n* = 289/976) among those with ischemic stroke. The highest refusal rate was observed among patients whose cause of death was classified as "other," a category predominantly comprising individuals with long-standing illnesses, with 70% of cases attributed to neoplastic or infectious diseases. Interestingly, there was a significant association between the time from event to death and donation outcome: among potential donors who died within 48 h, refusal rate was 27% (*n* = 2735/10046), whereas this proportion increased to 55% (*n* = 115/208) for those who died after more than 48 h (*p* < 0.001).

**Fig. 1:**
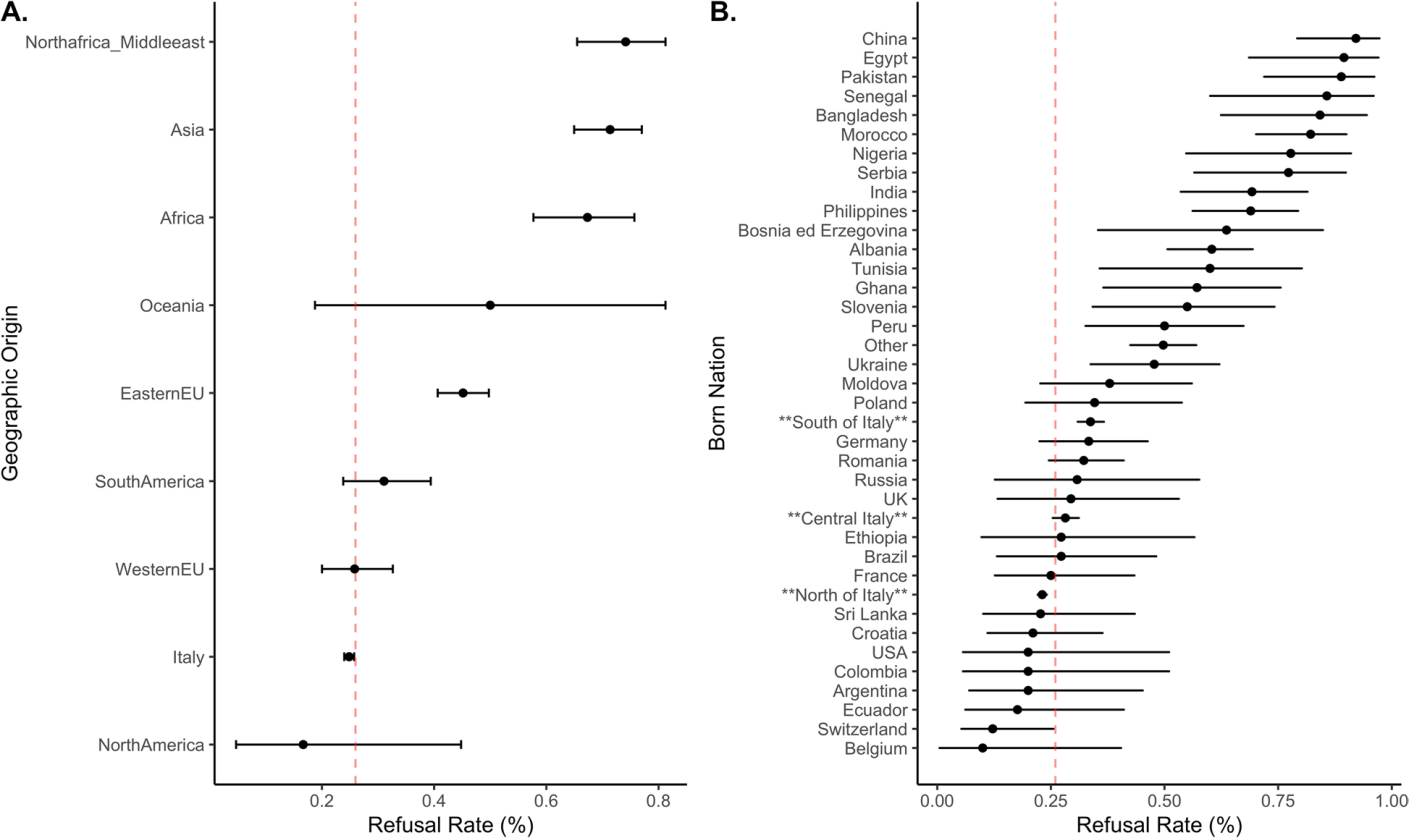
Caterpillar plot on differences in attitude towards organ donation between geographic origin (**A**) and country of birth (**B**), with the underlying refusal rate as the main outcome. In the right caterpillar plot we subdivided Italy into three main subgroups (north, central and south) to show intra-country variability

The birth geographic origin was associated with differences toward organ donation (*p*-value < 0.001). Significant variability was observed across geographical areas and countries, as shown in Fig. 1 and Table 1. Refusal rates ranged from 17% among North American subjects (albeit based on a small sample size) to 74% among those from North African and Middle Eastern origin. Overall, Italian-born patients presented a refusal rate of 25%, placing it in the lower range of refusal rate. Within the Italian-born subgroup there was a statistically significant upward trend in organ donation refusal rates from north to south (from 23 to 34% of refusal rate), as displayed in Figs. 1B and 3B.

### Multivariate analysis

**Fig. 2:**
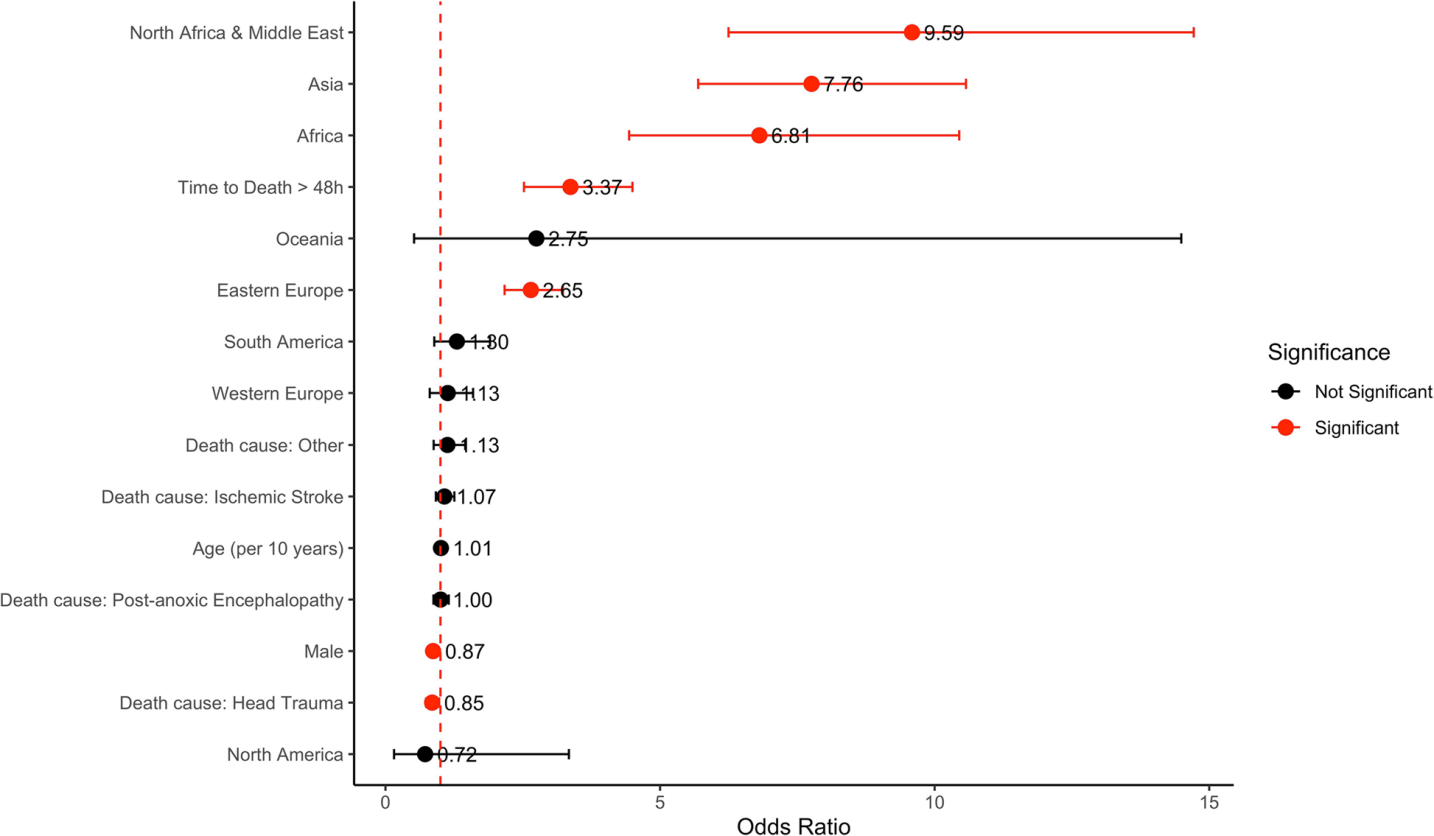
Fixed effects model showing odds ratios (OR) with 95% confidence intervals for various covariates. Odds ratios are plotted on a logarithmic scale. Red markers indicate statistically significant associations (*p* < 0.05), while black markers denote non-significant results

The multivariate logistic regression analysis identified several variables independently associated with family refusal of organ donation (Table 1; Fig. 2).

Male gender was significantly associated with lower likelihood of refusal compared to female gender (OR 0.87; 95% CI 0.79 – 0.95; *p* = 0.002). On the other hand, age was transformed into decades to improve model performance and interpretation but did not show a significant association with refusal.

**Fig. 3:**
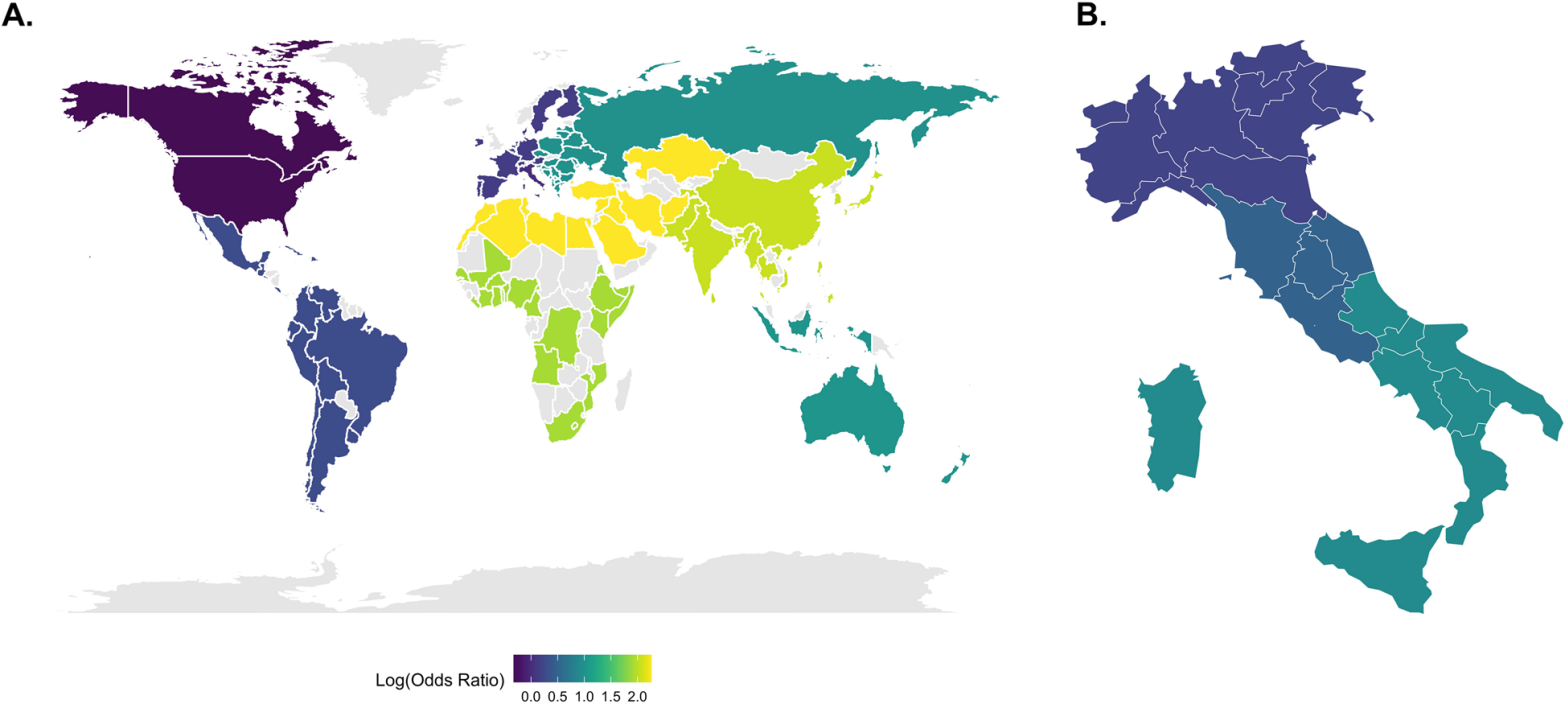
Variability of attitude towards organ donation stratified by geographic region of birth worldwide (**A**) and Italian subgroups (**B**), divided by Logarithmic Odds Ratio

The impact of donor geographic origin on refusal rates is represented by a world map showing the different geographic birth of the analysed donor population (Fig. 3A). We reported the odds ratios of the different population groups, compared with the reference population (Italy). Donor geographic origin were ranked from the highest to the lowest refusal rate: North Africa and Middle East (OR 9.59; 95% CI 6.25 – 14.72), Asia (OR 7.76; 95% CI 5.69 – 10.57), Africa (OR 6.81; 95% CI 4.44 – 10.45), Oceanian (OR 2.75; 95% CI 0.52 – 14.49), Eastern Europe (OR 2.65; 95% CI 2.17 – 3.23), South America (OR 1.65; 95% CI 1.15 – 2.38) and finally Western Europe (OR 1.13; 95% CI 0.81 – 1.59).

Regarding cause of death, only TBI was associated with a significantly lower risk of refusal compared to cerebral haemorrhage (OR 0.85; 95% CI 0.74–0.98; *p* = 0.023). Other causes, ischemic stroke and post-anoxic encephalopathy, were not independently associated with different refusal. Time between the event and death also had a notable impact. Refusal was more likely when death occurred more than 48 h after the initial event (OR 3.37; 95% CI 2.52–4.50; *p* < 0.001), compared to deaths within 48 h.

**Fig. 4:**
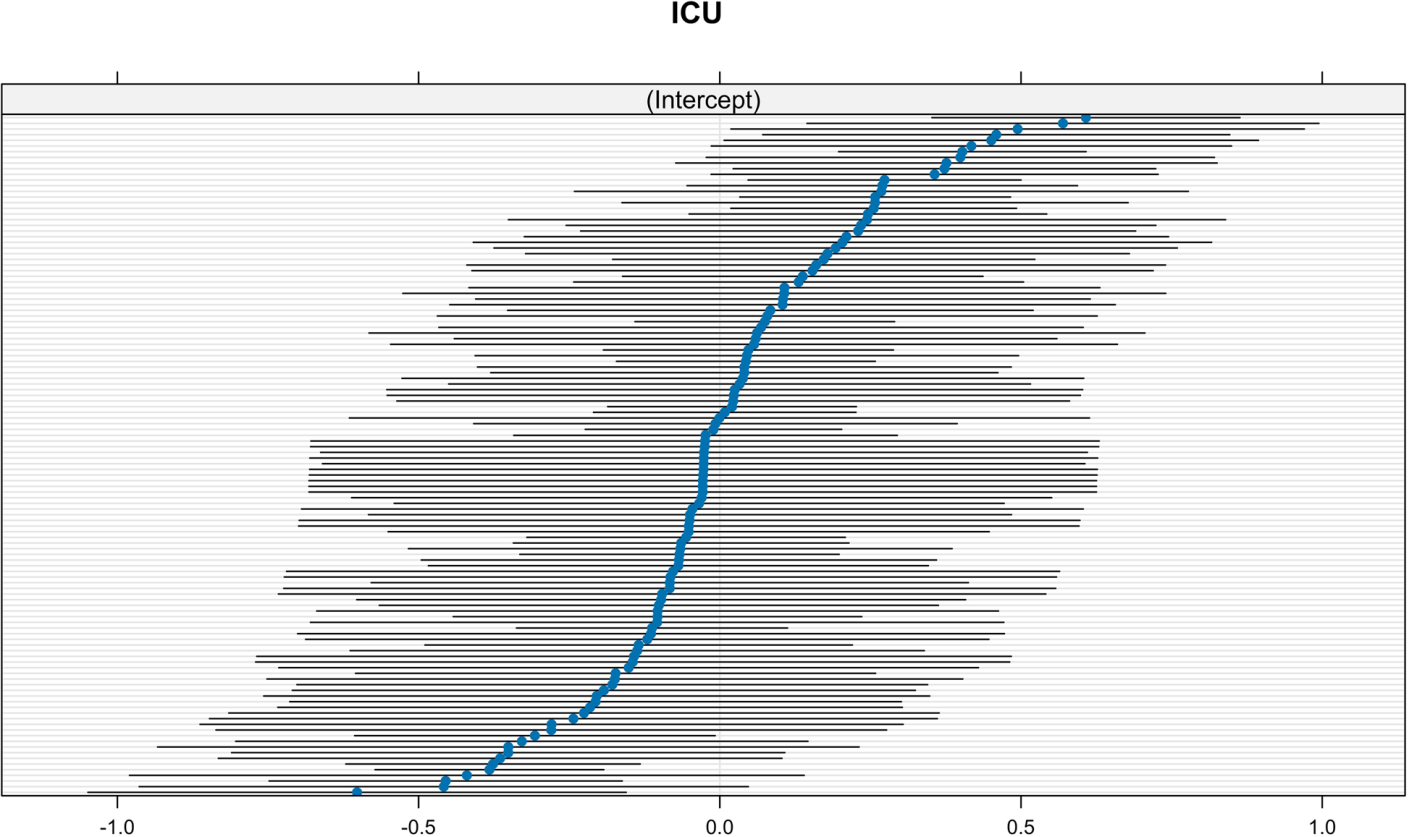
Median ICU-specific random intercepts with 95% confidence intervals from a mixed-effects model. Each point represents the estimated intercept for a specific intensive care unit (ICU), sorted by magnitude. The horizontal lines depict the associated uncertainty. ICUs with intercepts closer to + 0.5 suggest higher likelihoods of refusal, whereas ICUs with intercepts around –0.5 suggest lower refusal tendencies. This variation highlights the heterogeneity in clinical decision-making across centres, independent of fixed patient-level characteristics

In the multivariate multilevel model, the effect of individual ICUs was treated as a random effect. The median odds ratio (MOR) associated with ICU-level variability was 1.38, this means that, on average, the variability between different ICUs significantly increased the likelihood of refusal by approximately 38% (Fig. 4).

We therefore evaluated the only parameter available at the individual ICU level—its activity volume—by directly examining its association with the refusal rate. As illustrated in the scatterplot (Supplementary Material, Fig. 2S), notably refusal rates display substantial variability among ICUs with lower volumes. However, Pearson’s correlation analysis revealed only a weak positive association between ICU volume and refusal rate (*r* = 0.177; 95% CI: –0.002 to 0.345; *p* = 0.053), which did not reach statistical significance.

Finally, to evaluate the discriminatory ability of the multivariable model, we calculated the area under the receiver operating characteristic curve (AUC). The model yielded an AUC of 0.66, indicating a low to moderate ability to distinguish between cases and controls. To assess the internal validity of the model and estimate the stability of the AUC, we performed nonparametric bootstrapping with 1,000 resamples. The bootstrap results showed an average AUC of 0.65 with a negligible bias (–0.0004) and a standard error < 0.001 (ROC curve – Supplementary Material, Fig. 3S). The histogram of the bootstrapped AUCs approximated a normal distribution, supporting the robustness of the model’s discriminative performance.

### Population of Italian descent

The subpopulation of Italian donors (*N* = 9038—88% of the total population) was analysed separately with an overall refusal rate of 25%, which varied according to place of birth. The refusal rates in north, central and south Italy born, which stood at 23% (1627/9038 patients), 25% (253/942 patients), and 34% (351/1041 patients), respectively suggesting a statistically significant downward trend in consent to organ donation moving from north to south Italy (Table 1, Fig. 1B). In multivariate analysis, adjusted for demographic and clinical variables, and keeping the north of Italy as the reference level, central-Italy born had OR 1.34 (95% CI: 1.08—1.65; *p*-value 0.008) while the south-Italy born had OR 1.61 (95% CI: 1.39—1.86; *p*-value < 0.001), as shown in Fig. 3B.

## Discussion

Our results show that geographic origin, type of death, time from event to death and ICU-specific factors where consistently related with the rate of donation refusal.

### Strengths and weakness

Several potential factors influencing consent for deceased organ donation have already been analysed in literature including religious beliefs, cultural, social and systemic barriers, the practices and skills of ICU staff in shaping next of kin’s decisions, their knowledge about organ donation and the wishes of their loved one, as well as the cognitive and emotional impact of sudden loss [12]. Our results confirm observational studies that analyse the relevance of geographic origin on refusal rate in different countries (Spain, Norway, Great Britain) along Italy [2–9]. However, none of the previous studies address simultaneously the three main areas that could potentially influence organ donation approval: geographic origin, intrinsic factors related to ICUs, and intrinsic factors related to the family or donor. The strength of this study lies in the integration of these factors using a robust statistical model applied to a large population. This approach allowed us to identify the critical elements of the donation process while accounting for potential confounding factors.

Despite being comprehensive, our study has several limitations. While religious beliefs and variables specific to each cultural, social and systemic barrier are probably the real determinants of refusal rates, these variables were not collected prospectively. The term “ethnicity” is commonly defined as, “the social group a person belongs to, and either identifies with by others, as a result of a mix of cultural and other factors including language, diet, religion, ancestry, and physical features” [18]. However, since none of these variables were included in the Donor Manager registry, we used donor’s geographic origin – specifically the place of birth – as a proxy for ethnicity, in line with prior research [11].

### Main results

In the context of donor geographic origin, ICU physicians must be aware of and be prepared to face refusal rates exceeding 65% when dealing with African, Asian, or North African and Middle Eastern donors and their next of kin. This study shows that the relevance of geographic origin persists even when considering the influence of three other variables: cause of death, time from event to death and the intrinsic effect of the ICU. This suggestion aligns with broader literature that emphasizes the role of cultural and religious factors in shaping attitudes toward organ donation [2–9].

We also addressed potential intra-country barriers, revealing significant regional differences in authorization to organ donation within the Italian population, with an increasing trend of refusal from northern (23%) to southern Italy (34%). These variations may stem from cultural differences across Italy's diverse regions. This finding underscores the need for targeted strategies to address regional disparities, not only among foreign-born donors but also within Italy itself as already suggested by findings from a study conducted in the Turkish population [13].

Our findings also underline the potential influence of both the cause of death and timing from event to death. Consistent with existing literature, families appear more inclined to consent when death results from trauma [10], particularly in cases such as TBI. This may reflect a psychological need to find meaning in an otherwise senseless loss. However, a persistent barrier to consent remains the lack of acceptance of brain death, often rooted in hope for recovery or misunderstanding of the diagnosis. In line with prior surveys, denial of brain death emerged as a leading reason for refusal, alongside mistrust in the healthcare system and concerns about religious or bodily integrity [19].

Notably, while previous studies suggest that most decisions are made within 48 h, our data indicate that longer time intervals between the event and death correlate with higher refusal rates; this is opposed to findings from some retrospective analyses, which observed a modest increase in consent rates among families who had more time to process the situation. [20]. This discrepancy, together with the potential difficult understanding of brain death, may reflect contextual or cultural differences and highlights the need for sensitive, timely communication that provides families with both clarity and emotional support throughout the decision-making process.

Curiously, variability in ICU practices influencing family decisions has been previously documented and can be considered intrinsic to each ICU [12, 14–17]. An innovative finding of our study was the significant variability between different ICUs (up to 36%). This variability suggests that ICU-specific factors, possibly related to their ability to overcome cultural, systemic, and religious barriers, play a crucial role in influencing organ donation rates. Additionally, previous research [20] has shown that higher quality of ICU care, including end-of-life practices, is associated with lower standardized mortality rates and improved peri-mortem management. Our findings raise the possibility that similar ICU-specific quality factors may also influence donation outcomes, underscoring the need for further investigation into this area.

### Meaning of the study: possible implications for clinicians and policymakers

This study, by employing a robust multivariate multilevel model and leveraging a large cohort of patients, offers a predictive ability with an AUC of 66% the best achievable given the available variables. To our knowledge, no previous attempts have been made in the literature to develop a predictive model for organ donation refusal following death. While our model is statistically solid and supported by considerable sample size, it ultimately falls short of providing actionable individual-level predictions. This suggests that a predictive approach alone is insufficient. Instead, our findings point to the necessity of a comprehensive, integrated, and collaborative analysis that incorporates a broader set of ICU-specific and contextual variables to optimize the donation process. Therefore, three main areas of intervention could be identified. The first area represents a complex external factor that might be shaped by cultural beliefs, historical mistrust, and varying levels of awareness about organ donation across certain geographic areas. To address these barriers, targeted outreach and culturally sensitive education programs could be developed in collaboration with community leaders and stakeholders, aiming to foster trust and increase acceptance of organ donation within these populations. The second area of intervention could be to try to reduce the sense of responsibility experienced by the next of kin, regardless of ethnicity, by encouraging all persons to formally declare their own wishes, although this may not be necessarily in favor of donation. The third area involves the internal dynamics at individual ICUs, where refusal rates vary, suggesting that the environment and approach to discussing donation play a critical role. Interventions at the ICU level could include enhanced training in communication strategies for healthcare professionals, focusing on how to have clear, compassionate conversations with families about organ donation. It can be hypothesized that in some ICUs, donation refusal rate may be related to the overall quality of the ICU or, at least, the quality perceived by patients’ relatives [21]. Surprisingly, no observational studies have been conducted to examine how processes such as coherence, acceptance, and communication with next of kin influence perceptions of quality, build trust, and reduce refusal rates.

## Conclusions

Organ donation refusal emerges from the intersection of cultural context, family decision‐making and ICU‐level practices. While place of origin remains a strong marker of hesitancy, it interacts closely with whether donors or their next of kin have documented their wishes and with institutional processes that frame the consent discussion. Addressing this issue requires a coordinated, multi‐tiered approach that integrates culturally tailored community engagement, the systematic incorporation of specialized communication training for ICU personnel, and the establishment of iterative audit‐and‐feedback processes augmented by qualitative inquiry to identify and eliminate unit‐specific barriers. Only through this integrated approach we could meaningfully increase consent rates and fulfil the lifesaving promise of organ transplantation.

## Supplementary Information

Supplementary Material 1: Figure 1S. Flowchart of study population selection. Figure 2S. Scatterplot of ICU activity volume and refusal rate. Figure 3S. ROC curve of the multivariable model. Figure 4S. Flow diagram illustrating the process of grouping birth nations into macro groups.

## Data Availability

Researchers interested in accessing the data can contact the corresponding author to request the dataset in Excel format.

## Abbreviations

ICU: Intensive Care Unit
EU: European Union
NITp: North Italian Transplant program
