# Supplementary material for: Understanding organ donation refusal in the next of kin: a fifteen-year retrospective study in ten thousand potential donors
Source: J Anesth Analg Crit Care. 2025 Oct 9;5:60. doi: 10.1186/s44158-025-00282-7 (PMC12513025; doi:10.1186/s44158-025-00282-7)
Supplement: Supplementary file 1 — Supplementary Material 1: Figure 1S. Flowchart of study population selection. Figure 2S. Scatterplot of ICU activity volume and refusal rate. Figure 3S. ROC curve of the multivariable model. Figure 4S. Flow diagram illustrating the process of grouping birth nations into macro groups. [file 44158_2025_282_MOESM1_ESM.docx]

**Online Supplementary Material**

**Understanding Organ Donation Refusal: A Fifteen-year Retrospective Study.**

Stefano Marelli, Lorenzo Querci, Federico Pozzi, Cristiana Cipolla, Giuseppe Piccolo, Marco Sacchi, Tullia De Feo, Massimo Cardillo, Arturo Chieregato

**List of covariates available in the database**

1. Date of death = day, month and year of reported death.
2. Gender = binary variable expressed as male or female.
3. Cause of death = categorical variable related to the major cause associated with death.
4. Refusal to organ donation = binary variable expressed as permission or non-permission to donate an organ.
5. Geographic Origin = categorical variable which expresses the official country of birth by patient; ethnicity was derived from this variable, following the definition of ethnicity provided in the literature.
6. Time from event to death = numerical continuous variable representing the number of days between the main event and the declaration of death.
7. Volume of ICU = number of potential donors reported per specific ICU over a 15-year period.

**Figure 1S**

**Figure 1S**: Flowchart of study population selection. Among 12,930 registered potential donors, we excluded: 482 cases (3.7%) due to evident ineligibility for donation, 4 cases (<0.01%) with missing information on place of birth or donation wishes, 684 donors (5.5%) who donated via circulatory death (DCD), and 1,506 donors (12.8%) who had formally expressed their wishes during their lifetime. The final study population included 10,254 potential donors.

**Figure 2S**


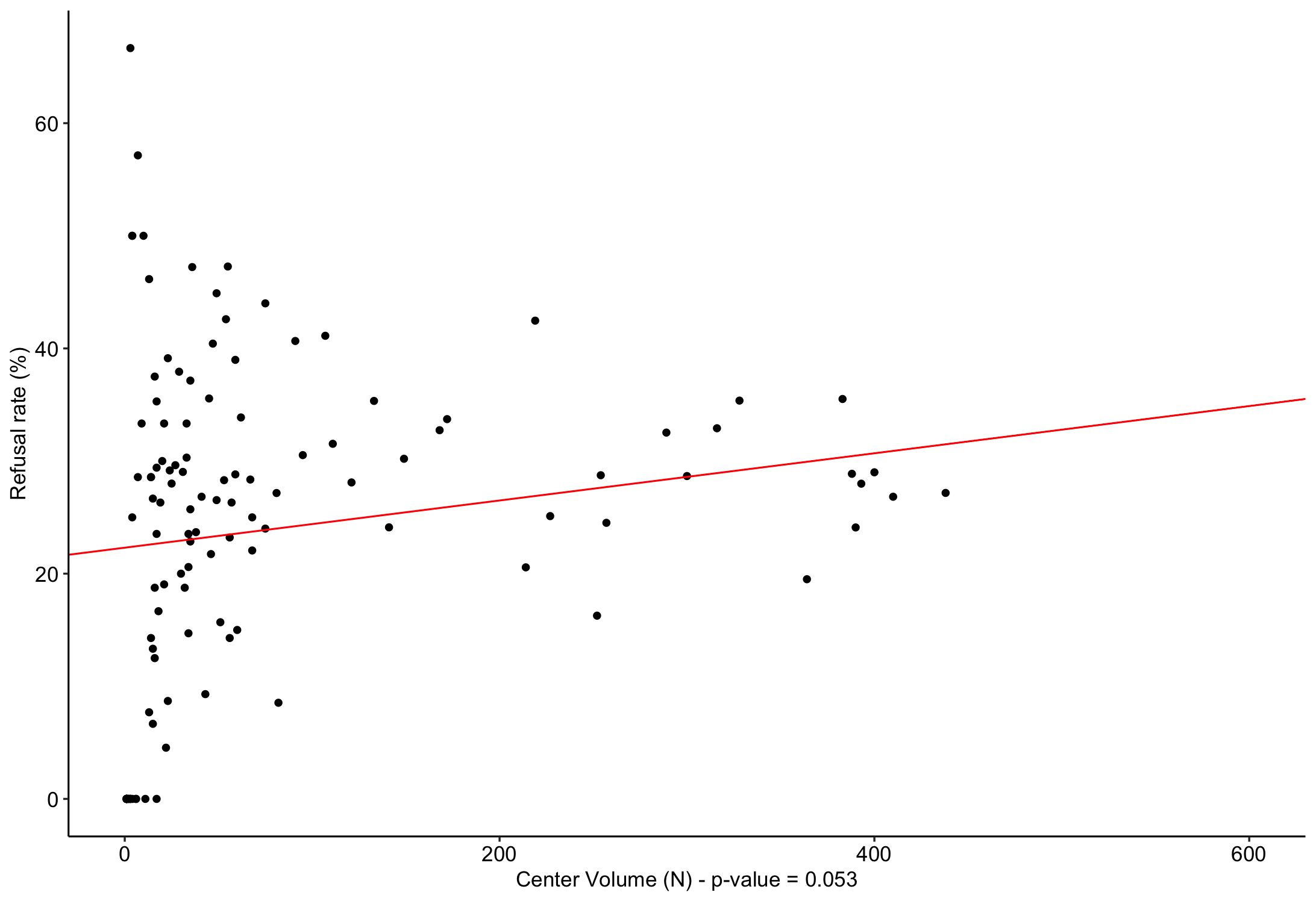


**Figure 2S**: Scatterplot of ICU activity volume and refusal rate. Each point represents an individual ICU, with refusal rate plotted against the total number of potential donors managed. The red line indicates the linear regression trend. Refusal rates display substantial variability at lower ICU volumes. Pearson’s correlation showed a weak positive association (r = 0.177; 95% CI: –0.002 to 0.345; p = 0.053), which did not reach statistical significance.

**Figure 3S**


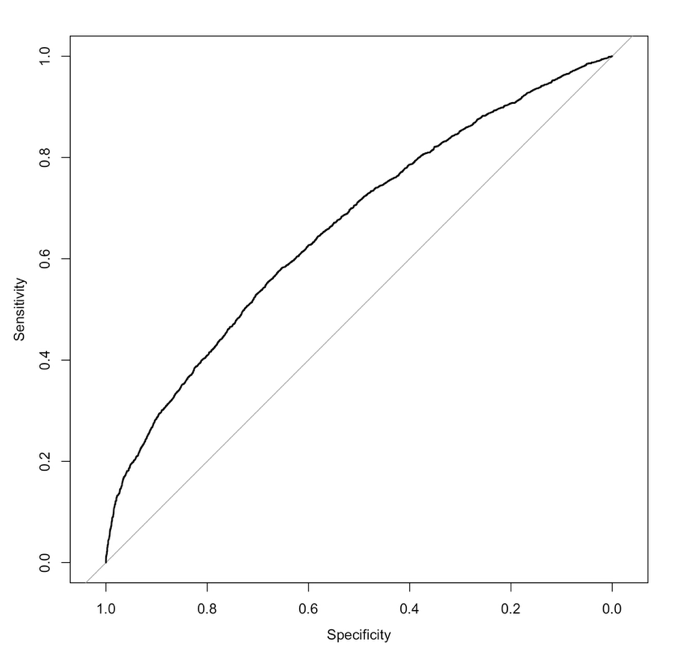


**Figure 3S**: Receiver Operating Characteristic (ROC) curve of the multivariable model. The area under the curve (AUC) was 0.66, indicating a moderate discriminative ability to distinguish between cases and controls. This ROC curve reflects the model's performance in the original dataset prior to internal validation via bootstrapping.

**Figure 4S**

**Figure 4S**: flow diagram illustrating the process of grouping birth nations into macro groups to produce eight areas of donor geographic origin (excluding Italy).
